# One-Way and Two-Way Mobile Phone Text Messages for Treatment Adherence Among Patients With HIV: Protocol for a Randomized Controlled Trial

**DOI:** 10.2196/16127

**Published:** 2020-09-30

**Authors:** Dickson Shey Nsagha, Vincent Verla Siysi, Same Ekobo, Thomas Obinchemti Egbe, Odette Dzemo Kibu

**Affiliations:** 1 Department of Public Health and Hygiene University of Buea Buea Cameroon; 2 Department of Medicine University of Buea Buea Cameroon; 3 Department of Obstetrics and Gynecology University of Buea Buea Cameroon

**Keywords:** HIV, antiretroviral therapy, short message service, adherence

## Abstract

**Background:**

Incomplete adherence to antiretroviral therapy (ART) is one of the factors that contribute to HIV drug resistance, and it is a major problem for the public health system in controlling the HIV pandemic. There is emerging evidence that SMS can play an important role in health care delivery among patients with HIV on ART, especially in resource-limited settings.

**Objective:**

This paper aims to assess the impact of two-way and one-way SMS text messaging on adherence to HIV treatment. We hypothesized that sending weekly text messages through the one-way and two-way SMS text messaging approach will improve adherence to ART among patients with HIV and improve associated clinical outcomes (quality of life).

**Methods:**

A randomized controlled trial is being carried out among participants with HIV who have been on ART for at least one month from an accredited treatment center, namely the Buea Regional Hospital and Kumba District Hospital of South West Region, Cameroon. Participants with HIV, both male and female, aged 21 years and older make up a sample size of 207. The interventions involved the use of mobile phone text messages. Before commencing the intervention, a focus group discussion was carried out among the participants to understand their perception about the use of SMS-based interventions to improve adherence. A total of 246 participants were randomized to receive either a one-way text message (SMS sent to a recipient without recipient sending a reply) or two-way text message (SMS sent to a recipient and recipient sends a reply) or the control (no SMS, only standard care). Data on adherence and quality of life were collected at baseline and after 6 months and will be analyzed using SPSS version 21, while qualitative data will be analyzed using Atlas.ti 7.5.

**Results:**

Data collection began in September 2019 with focus group discussions and baseline data collection. After 1 month of baseline data collection, the intervention began in October 2019, and postintervention data were collected after 6 months (March 2020). At the end of the study, we will be able to understand the perception of patients toward SMS text messaging–based interventions and also assess the impact of one-way and two-way SMS text messages on treatment adherence among patients with HIV and on associated clinical outcomes (quality of life).

**Conclusions:**

The impact of SMS text messaging varies across different settings. The results from this study will determine the perception of patients toward an SMS text messaging–based intervention and its impact on adherence to ART.

**International Registered Report Identifier (IRRID):**

DERR1-10.2196/16127

## Introduction

Medication adherence is defined by the World Health Organization as the degree to which a person's behavior corresponds with the agreed recommendations from a health care provider or as the extent to which the patient's history of therapeutic drug taking coincides with the prescribed treatment. The Joint United Nations Programme for HIV and AIDS (UNAIDS) has deﬁned new ambitious targets that call for 90% of people living with HIV to know their status, 90% of those diagnosed to receive antiretroviral therapy (ART), and 90% of those on treatment to achieve an undetectable viral load. This is known as the 90-90-90 target [1]. In 2018, 37.9 million people were living with HIV worldwide [2]. Scale-up of antiretroviral therapy is on a fast-track trajectory that has surpassed expectations. The global coverage of antiretroviral therapy reached 46% (43%-50%) at the end of 2015 [1]. In sub-Saharan Africa, where the majority of people receiving antiretroviral therapy live, timely access to HIV diagnosis and linkage to care remain the main challenges to achieving the 90-90-90 objective. In 2018 in Cameroon, 74% of people living with HIV knew their status, and only 52% of people living with HIV were on treatment [2].

Incomplete adherence to antiretroviral therapy is one of the factors that contribute to HIV drug resistance, HIV disease progression, and death [3,4]. Most patients do not adhere to ART due to the long duration of HIV treatment (lifelong therapy) and associated side effects [5]. Diverse strategies have been tested and implemented to help improve adherence to ART.

There is emerging evidence that mobile phones can play an important role in health care delivery, especially in resource-limited settings [6]. The use of phones to promote adherence has grown as phone ownership rates continue to rise in sub-Saharan Africa and elsewhere [7,8]. SMS text messaging is a particularly useful application that can be used to collect or share information and to enhance communication between health care personnel and patients in a low-cost manner [9]. With regard to patient management, mobile phone text messages have been demonstrated to induce positive behavior changes in domains such as smoking cessation, physical activity, and self-management of high blood pressure, diabetes, and asthma [10]. Other studies report high levels of satisfaction among participants [11,12]. Considering these studies, it is possible to conclude that SMS is effective in inducing a positive behavior change and providing greater connectedness with a provider.

Some studies have shown the effectiveness of SMS in improving adherence. For example, a recent Cochrane systematic review synthesizing data from 2 trials conducted in Kenya by Lester and colleagues [12] showed that text messaging is efficacious in improving adherence. They tested a two-way SMS intervention in which recipients in the intervention group were required to respond to weekly messages inquiring about their well-being within 48 hours, with those indicating that they did not feel well or not responding at all receiving outreach from clinic staff. They found a 12 percentage point increase in the likelihood of self-reported adherence greater than 95% among HIV-positive adults in 3 clinics, as well as a 9 percentage point increase in rates of viral suppression. The use of SMS text messaging was also shown to be effective by Pop-Eleches and colleagues [13]. They reported a 13 percentage point increase in the likelihood of achieving at least 90% electronically monitored adherence over 48 weeks in the intervention group that received one-way weekly SMS messages. In another study, Hailey and Arscott [14] showed a significant increase in adherence rates (40% to 50% at baseline to 80% after 24 months) among youths living with HIV after sending a text message reminder and receiving a reply over a 24-month period. However, the youths did not send text messages about their drug refills, shortages of their medications, and adverse effects of the medications they were receiving. Due to the fact that this intervention prevented the patients from sending text messages about adverse effects and the state of their well-being, it is possible to question their quality of life and its impact on adherence. Though SMS text messaging can increase adherence rate, poor quality of life can affect adherence if the content of the text does not address issues related to quality of life.

A study carried out in Cameroon reported a contrary view on SMS text messaging improving adherence [15]. In addition, another study carried out by Linnemayr and colleagues [16] also reported the ineffectiveness of SMS text messaging in improving adherence.

However, despite the quality of evidence generated from literature [10-14] on the importance of SMS-based interventions in improving adherence treatment, especially ART, it remains unclear if the efficacies observed in this existing literature translate into effectiveness in different settings. The fact that a text messaging intervention is effective in a particular setting does not signify its effectiveness in other settings. Some text message–based interventions are ineffective because researchers did not understand or take into consideration the conditions under which the SMS text messaging intervention should be implemented and the content of the message. It is also important to find out what aspect of behavior change the text messaging affects and how it can generally cause behavior change. The findings from this study will be used by health care providers to effectively design an SMS text messaging intervention that is applicable to the setting and the target population.

The general objective of this study will be to assess the impact of the interventions (two-way and one-way SMS) on adherence to ART. We hypothesize that sending weekly text messages through the one-way and two-way SMS text messaging approach will improve adherence to ART among patients with HIV and improve associated outcomes (quality of life).

## Methods

### Study Setting and Participants

The participants were selected from accredited treatment centers, namely the Buea Regional Hospital and Kumba District Hospital of South West Region, Cameroon. Participants included adults (male and female) aged 21 years and older who were infected with HIV and visiting the health centers for routine health education and drug refill appointments. The study recruited participants that were on highly active antiretroviral therapy (HAART) for at least 1 month and who had mobile phones and were able to read and send text messages. We excluded pregnant women and patients with drug-resistant HIV prior to the commencement of the study.

### Study Design

A qualitative study design involving the use of focus group discussions (FGDs) was used to assess the perception of patients on the use of SMS text messaging–based intervention. Thereafter, a quantitative study design involving a randomized controlled trial was carried out. The intervention used one-way or two-way SMS text messaging. Adherence and quality of life (QoL) were measured at baseline and at postintervention in control and intervention groups. Adherence in the control group will be compared with the adherence in the intervention groups before and after the intervention. Likewise, the QoL in the control group will be compared with the QoL in the intervention groups before and after the intervention. In addition, the impact of adherence on QoL will be assessed in the control group and intervention groups before and after the intervention.

### Intervention

**Figure 1:**
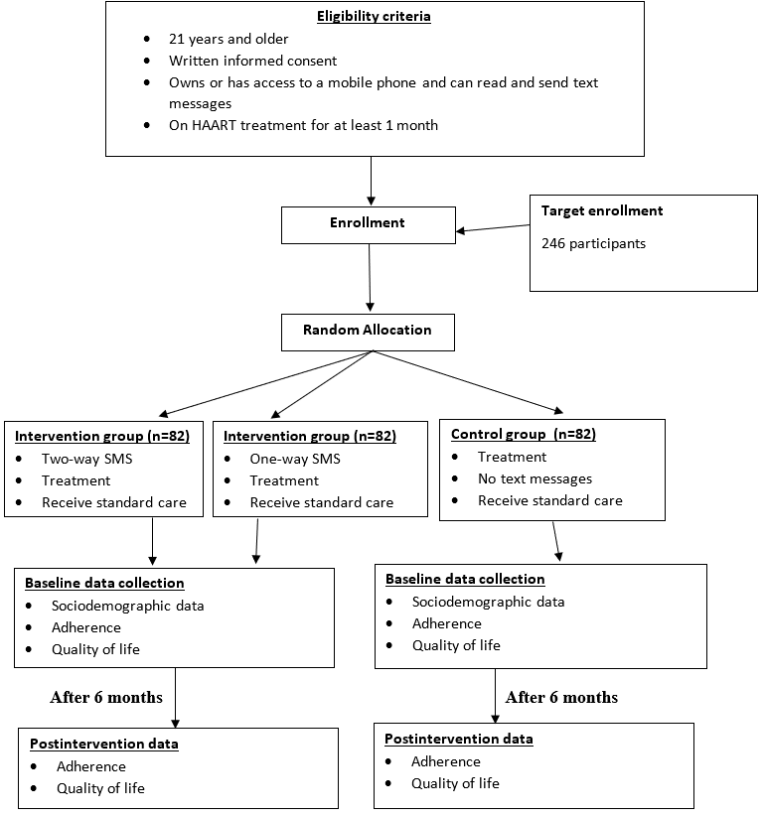
Flow diagram on the randomization of participants into the intervention and control groups. HAART: highly active antiretroviral therapy.

The interventions involved the use of mobile phone text messages in addition to the standard care provided to the participants. Participants were randomized into either a one-way text message group (text message sent without receiving a reply) or two-way text message group (text message sent to recipients and the recipient replies to the SMS sent). Text messages were sent both in English and in French, and airtime was purchased from the mobile telephone network. In the morning periods, messages were sent 3 times a week (Mondays, Wednesdays, and Fridays between 8 AM and 10 AM) from an established list of phone numbers of the participants during the study period. The control group received only the standard care given at the hospital. The random allocation of participants in the intervention and control group is shown in Figure 1. The intervention used in this study is based on the nudge theory, which is an approach to understanding and changing people's behavior by analyzing, improving, designing, and offering free choices for people so that their decisions are more likely to produce helpful outcomes for those people and society generally. The SMS text messaging acts as a nudge in order to influence a positive behavior change, which is adherence to ART.

### Study Duration

This study will be carried out over a period of 1 year. The intervention phase ran for 6 months after collecting baseline data (preintervention data) on adherence and quality of life.

### Sample Size

The formula by Chan [17] for calculating the sample size for randomized controlled trials was used:


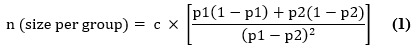


In the above equation, *c* = 7.9 for 80% power and *p*1 and *p*2 are the proportion estimates (60% for the control group and 82% for the intervention group).


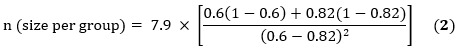


n (size per group) = 63.27 **(3)**

It was assumed that 30% of the participants would drop out of the study due to loss to follow-up and mortality. A total of 82 participants were then randomized to each group to allow for the 30% drop out (30% of 63 participants = 19). In order to make up for the 19 that was assumed to be lost to follow-up, we added the 19 participants to the calculated 63 participants, giving a total of 82 participants. Therefore, a minimum of 246 participants were recruited into the study.

The above sample size in each group was distributed with 164 in the intervention group (82 in the two-way SMS group and 82 in the one-way SMS group) and 82 in the control group.

### Evaluation of Intervention

A logbook was used to register all SMS text messages sent and replies received on the designated days of the week. A delivery report function was used to ensure that the messages were delivered to the participants. The sent text and responses received will be evaluated by calculating the proportion of SMS text messages sent and responses received. Example text messages are shown in Textbox 1.

**Examples of the text messages that were sent.**

- “Hello. Do remember to take your medication as prescribed. Your health is your wealth.”
- “You are kindly reminded to take your medication. Your health is our priority.”
- “Don’t forget to take your medications.”

### Randomization

Using a simple randomization technique, participants were recruited into the intervention and control group. Allocation of the participants into the groups (intervention or control) was concealed during the randomization procedure. This shall be a double-blind controlled trial: data collectors and the statistician shall be blinded to the different group allocations.

### Data Collection Tools

A pretested structured questionnaire was used to obtain information from the participants on sociodemographic characteristics, adherence to treatment, quality of life, and past health history. A logbook was used to report on all messages that were sent during the week and the number of replies received, and the research team recorded all defaulters and participants lost to follow-up.

#### Measurement of Adherence

The overall adherence of each participant was measured using 2 scales of self-report, namely the visual analog scale (VAS) [18] and the Center for Adherence Support Evaluation (CASE) Adherence Index [19]. VAS appears to be a simple and easy to use measure of adherence, though there is mixed literature about this. VAS has been shown to have large strength associations with most other measures of adherence. A 100-point VAS will be used, with 0 indicating “absolute nonadherence” and 100 indicating “excellent adherence” in the last 30 days. A VAS score equating to at least 95% will be defined as optimal adherence and VAS scores less than 95% will be considered suboptimal.

The CASE Adherence Index is a simple composite measure of self-reported ART adherence. Based on the results of correlation and principal components analyses, the CASE Adherence Index is developed as a composite (sum) of 3 self-reported measures of adherence. The CASE Adherence Index can be seen in Multimedia Appendix 1.

#### Measurement of Quality of Life

The quality of life was assessed using the abbreviated World Health Organization Quality of Life (WHOQOL-BREF). This is an international, cross-culturally comparable quality of life assessment instrument. It assesses the individual's perceptions in the context of their culture and value systems and their personal goals, standards, and concerns. The WHOQOL-BREF instrument comprises 26 items, which measure the following broad domains: physical health, psychological health, social relationships, and environment [20]. The quality of life among the participants with HIV was assessed using the WHOQOL-HIV BREF. The WHOQOL-HIV BREF is based on the WHOQOL-BREF, the shorter form of the WHOQOL-100. This contains 5 extra items specific to people living with HIV/AIDS and in total contains 31 items [21].

#### Focus Group Discussion

A FGD discussion guide was pretested and fine-tuned before implementing it in the study. A total of 8 FGDs will be organized under 4 main themes: (1) predictors of nonadherence to ART, (2) awareness of the use of mobile health (SMS) to improve adherence to ART, (3) content of the text that will be acceptable for use to improve adherence to ART, (4) choice of approach to text messaging services (one-way or two-way), and (5) possible challenges in the implementation of mobile health (via SMS). All FGD sessions will last for 50 to 60 minutes and be moderated by a moderator. At the end of the study, a FGD will be conducted to discuss the challenges the participants faced with respect to text message reminders and replies.

### Study Outcome

#### Primary Outcome

The primary outcome is to assess the impact of the one- and two-way SMS text messages on treatment adherence among patients with HIV. Measures of adherence from the CASE Adherence Index and the VAS will be used to measure the overall adherence.

#### Secondary Outcome

The secondary outcome is to assess the impact of the intervention on quality of life and the impact of quality of life on adherence.

### Data Management and Statistical Analysis

Data from the questionnaires will be checked each time they are brought from the field for unfilled and unanswered questions, including the verification of codes. All verified data and codes will be keyed into Microsoft Excel and research questionnaires will be kept in a secured locker. The analysis of patient demographics and study outcome variables will be summarized using descriptive summary measures, expressed as mean (standard deviation) or median (minimum-maximum) for continuous variables and number (percentages) for categorical variables. A 1-tailed *t* test shall be used for comparing groups on continuous outcomes, and the chi-square test will be used for binary outcomes, as shown in Table 1. Analysis of variance will be used to analyze the statistical differences among more than two group means. All statistical tests will be performed using 2-sided tests at the .05 level of significance. All statistical analyses will be performed using SPSS (version 21.0; IBM Corp).

**Table 1:** Statistical tests to be used for different variables.

Outcome variables			Scale	Type	Measure	Analysis method
**Primary**						
	**Adherence at baseline and at 6 months**					
		CASE^a^ Adherence Index	Ordinal	Binary	Adherence in last month ≥95%	Chi-square test
		VAS^b^	Ordinal	Binary	VAS percentage ≥95%	Chi-square test
**Secondary**						
	Quality of life		Ordinal	Categorical	Change in QoL^c^ scores	*t* test ANOVA^d^

^a^CASE: Center for Adherence Support Evaluation.

^b^VAS: visual analog scale.

^c^QoL: quality of life.

^d^ANOVA: analysis of variance.

### Ethical Considerations

Ethical approval for this study has been obtained (No. 2018/764-03/UB/SG/IRB/FHS) from the Faculty of Health Sciences Institutional Review Board of the University of Buea, including an administrative authorization from the South West Regional Delegation of Public Health, Cameroon. In this study, patients have voluntarily confirmed their participation in the study. They were enrolled in the study upon receipt of signed informed consent from the participant. Names and national identity card numbers shall not be used to identify the participants, but rather, codes shall be used. This will keep the participants anonymous. Secondly, in order to prevent social stigma, the mobile phone text message reminders will not disclose a participant's seropositive status.

## Results

Data collection began in September 2019 with focus group discussions and baseline data collection. After 1 month of baseline data collection, the intervention began in October 2019 and postintervention data were collected after 6 months (March 2020). At the end of the study, we will be able to assess the perception of patients toward SMS-based interventions and also assess the impact of the one- and two-way text messaging on treatment adherence among participants with HIV and on associated outcomes (quality of life). At baseline, the adherence and QoL in the control group will be compared with adherence and QoL in the intervention group, as well as at postintervention. Thus, the baseline results will be compared with the postintervention data to determine the impact of the intervention on adherence and on QoL. Adherence will be compared with QoL across all the groups at baseline and at postintervention.

## Discussion

### Overview

Despite the incredible success of the large-scale public sector provision of ART to HIV-infected people, failure to retain these patients on long-term treatment threatens to undermine the massive gains made since 2004 and remains one of the most critical obstacles to achieving the UNAIDS 90-90-90 targets for 2020, which are a key milestone toward ending the HIV epidemic by 2030 [22]. Diverse strategies or interventions have been put in place to improve adherence to ART. Research has shown that very high levels of adherence are required to obtain the maximum benefit of HAART. This situation justifies the importance of developing efficient strategies to improve adherence to ART [23].

There is emerging evidence that mobile phones can play an important role in health care delivery, especially in resource-limited settings [6]. The appeal of using phones to promote adherence has grown as phone ownership rates continue to rise in sub-Saharan Africa and elsewhere [8]. SMS text messaging is a particularly useful application that can be used to collect or share information and to enhance communication between health personnel and patients in a low-cost manner [9]. With regard to patient management, mobile phone text messages have been demonstrated to induce positive behavior changes in domains such as smoking cessation, physical activity, and self-management of high blood pressure, diabetes, and asthma [10]. Other studies report high levels of satisfaction among participants [11,12]. With Africa currently undergoing a digital revolution, the World Health Organization Regional Office for Africa and the International Telecommunication Union signed a cooperation agreement in Geneva on October 26, 2017, the focus of which was on how best to use digital services to save lives and improve people’s health [24].

The intervention in this study uses the health belief model of behavior change [25]. The data on the perceived barriers to adherence will be collected (through FGDs) and, as a cue to action for patients to adhere to treatment, the efficacy of text message reminders will be tested.

### Conclusion

The impact of SMS text messaging varies across different settings. The results from this study will determine the perception of patients toward text message–based interventions and the impact of text messaging interventions on health care delivery among patients with HIV on ART.

## Appendix

Multimedia Appendix 1 The CASE Adherence Index.

## Abbreviations

ART: antiretroviral therapy
CASE: Center for Adherence Support Evaluation
FGD: focus group discussions
HAART: highly active antiretroviral therapy
QoL: quality of life
UNAIDS: Joint United Nations Programme for HIV and AIDS
VAS: visual analog scale
WHOQOL-BREF: abbreviated World Health Organization Quality of Life
