# Supplementary material for: One-Way and Two-Way Mobile Phone Text Messages for Treatment Adherence Among Patients With HIV: Protocol for a Randomized Controlled Trial
Source: JMIR Res Protoc. 2020 Sep 30;9(9):e16127. doi: 10.2196/16127 (PMC7557438; doi:10.2196/16127)
Supplement: Multimedia Appendix 1 [file resprot_v9i9e16127_app1.docx]

**CASE Adherence Index**

(1) Frequency of difficulty taking HIV medications on time

A1: Self-reported frequency of ‘difficulty taking HIV medications on time (no more than two hours before or two hours after the time your doctor told you to take it)’.

**Responses:** 1) never, 2) rarely, 3) most of the time, 4) all of the time.

(2) Average number of days per week that HIV medication dose was missed

A2: Self-reported ‘average number of days per week at least one dose of HIV medications was missed’.

**Responses:** 1) every day, 2) 4–6 days per week, 3) 2–3 days per week, 4) once a week, 5) less than once a week, 6) never.

(3) Last time an HIV medication dose was missed.

A3: Self-reported ‘last time missed at least one dose of HIV medications’.

**Responses:** 1) within the past week, 2) 1–2 weeks ago, 3) 3–4 weeks ago, 4) between one and three months ago, 5) more than three months ago, 6) never.

The sum of the numbers next to each of the three questions gives the CASE index score.

INDEX SCORE: _________

> 10 = good adherence

< 10 = poor adherence
